# Compensation of Respiratory-Related Postural Perturbation Is Achieved by Maintenance of Head-to-Pelvis Alignment in Healthy Humans

**DOI:** 10.3389/fphys.2019.00441

**Published:** 2019-04-24

**Authors:** Valérie Attali, Louis Clavel, Philippe Rouch, Isabelle Rivals, Ségolène Rémy-Néris, Wafa Skalli, Baptiste Sandoz, Thomas Similowski

**Affiliations:** ^1^INSERM, UMRS1158 Neurophysiologie Respiratoire Expérimentale et Clinique, Sorbonne Université, Paris, France; ^2^Service des Pathologies du Sommeil (Département “R3S”), Hôpitaux Universitaires Pitié Salpêtrière – Charles Foix, Assistance Publique Hôpitaux de Paris (APHP), Paris, France; ^3^Arts et Metiers ParisTech, Institut de Biomécanique Humaine Georges Charpak (IBHGC), Paris, France; ^4^Equipe de Statistique Appliquée, ESPCI Paris, PSL Research University, Paris, France; ^5^Médecine Intensive et Réanimation (Département “R3S”), Hôpitaux Universitaires Pitié Salpêtrière – Charles Foix, APHP, Paris, France

**Keywords:** biplanar X-ray, personalized 3D models of the spine, lung volume, postural alignment, cervical and pelvic compensatory mechanisms

## Abstract

The maintenance of upright balance in healthy humans requires the preservation of a horizontal gaze, best achieved through dynamical adjustments of spinal curvatures and a pelvic tilt that keeps the head-to-pelvis alignment close to vertical. It is currently unknown whether the spinal and pelvic compensations of respiratory-related postural perturbations are associated with preservation of the head-to-pelvis vertical alignment. We tested this hypothesis by comparing postural alignment variables at extreme lung volume (total lung capacity, TLC; residual volume, RV) with their reference value at functional residual capacity (FRC). Forty-eight healthy subjects [22 women; median age of 34 (26; 48) years] were studied using low dose biplanar X-rays (BPXR; EOS^®^system). Personalized three-dimensional models of the spine and pelvis were reconstructed at the three lung volumes. Extreme lung volumes were associated with changes of thoracic curvature bringing it outside the normal range. Maximal inspiration reduced thoracic kyphosis [T1–T12 angle = 47° (37; 56), -4° variation (-9; 1), *p* = 0.0007] while maximal expiration induced hyperkyphosis [T1–T12 angle = 63° (55; 68); +10° variation (5; 12), *p* = 9 × 10^-12^]. Statistically significant (all *p* < 0.01) cervical and pelvic compensatory changes occurred [C3–C7 angle: +4° (-2; 11) and pelvic tilt +1° (0; 3) during maximal inspiration; C3–C7 angle: -7° (-18; -1) and pelvic tilt +5° (1; 8) during maximal expiration], resulting in preserved head-to-pelvis alignment (no change in the angle between the vertical plane and the line connecting the odontoid process and the midpoint of the line connecting the center of the two femoral heads ODHA). Lung volume related postural perturbations were more marked as a function of age, but age did not affect the head-to-pelvis alignment. These findings should help understand balance alterations in patients with chronic respiratory diseases that modify lung volume and rib cage geometry.

## Introduction

The maintenance of upright balance in healthy humans largely depends on the preservation of an horizontal gaze (Hasegawa et al., 2017). In this respect, achieving optimal balance with minimal energy expenditure implies keeping the head-to-pelvis alignment near vertical (Vital and Senegas, 1986; Dubousset, 1994; Amabile et al., 2018). Spinal curvatures and pelvic tilt are continuously adjusted to this effect, on the basis of information transmitted to the central nervous system via visual and proprioceptive afferents (Perennou et al., 2008; Amabile et al., 2016).

Although seldomly integrated into models describing human balance and spinal stability (Granata and Wilson, 2001), the rib cage imposes important postural adjustment constraints. Costovertebral joints limit flexion (especially lateral) and rotation of the thoracic spine (Liebsch et al., 2017) and their mechanical properties modulate the force exerted on the upper lumbar spine during trunk flexion (Ignasiak et al., 2016). In addition, breathing involves rotation of the costovertebral joints that modifies spinal curvature and, consequently, spinal postural alignment (Dally, 1908). It ensures that breathing induces a cyclic postural perturbation. This is reflected by respiratory-induced oscillations of the center of pressure, defined as the projection to the ground of the barycenter of vertical reaction forces, distributed over the entire surface of foot-ground contact. These oscillations disappear during breath-holding (Caron et al., 2004). They increase when ventilation increases (David et al., 2012).

The postural perturbations related to breathing in healthy subjects are centrally integrated and cyclically compensated by variations of spinal muscular rigidity (phasic contractions of paravertebral muscles), ensuring the maintenance of balance (Kantor et al., 2001; Hamaoui et al., 2010). Pelvic adaptations, consisting of phasic contractions of pelvic floor muscles synchronous with diaphragmatic contractions (Hodges et al., 2007; Talasz et al., 2011) and “respiratory” changes of lumbopelvic and hip angles (Grimstone and Hodges, 2003), are also involved in cyclic compensation of breathing-related postural perturbations. Whether or not the spinal and pelvic compensations of respiratory-related postural perturbations are associated with preservation of the head-to-pelvis vertical alignment (as a general balance maintenance mechanism) is currently unknown. The present study was designed to test the hypothesis that this is indeed the case, namely that head-to-pelvis vertical alignment is not affected by lung volume. To this purpose, we used the EOS^®^imaging device, a low-dose irradiation biplanar X-ray system (BPXR) (Dubousset et al., 2008) validated for the three-dimensional description of the normal and pathological weight bearing spine (Ilharreborde et al., 2013). With this device, we describe postural alignment and its adjustments as a function of lung volume in upright healthy volunteers. To maximize the effects of lung volume on spinal geometry (and therefore to test our hypothesis under the most extreme compensatory conditions), we performed our measurements over the full range of vital capacity (VC), namely at residual volume (RV, end of a maximal expiration) and total lung capacity (TLC, end of a maximal inspiration), using functional residual capacity (FRC, end-expiratory relaxation volume) as our reference point.

## Materials and Methods

### Subjects

Fifty healthy subjects [22 women, 28 men, 34 (26; 48) years, Body Mass Index 24 (21; 26) kg/m^2^] with no signs of postural dysfunction on clinical examination and normal pulmonary auscultation and pulmonary functional tests, were included. This study was approved by the *Comité de Protection des Personnes Ile-de-France VI* (Ethics Committee) on February 18, 2015 and is registered in the ISRCTN registry under number ISRCTN56129394. All patients provided their written informed consent.

### Reference Spirometric Values

Prior to the biplanar X-ray acquisitions (BPXR), pulmonary function tests were performed according to recommended standards (Wanger et al., 2005), using a spirometer to measure vital capacity (VC) and the helium dilution technique to measure FRC and calculate residual volume (RV, equal to FRC minus expiratory reserve volume -ERV-) and total lung capacity (TLC, equal to FRC plus inspiratory capacity -IC-) (Brown et al., 1998). The values of IC and ERV measured during pulmonary function testing are noted IC_pft_ and ERV_pft_, respectively.

### Lung Volumes During BPXR Acquisitions

**FIGURE 1:**
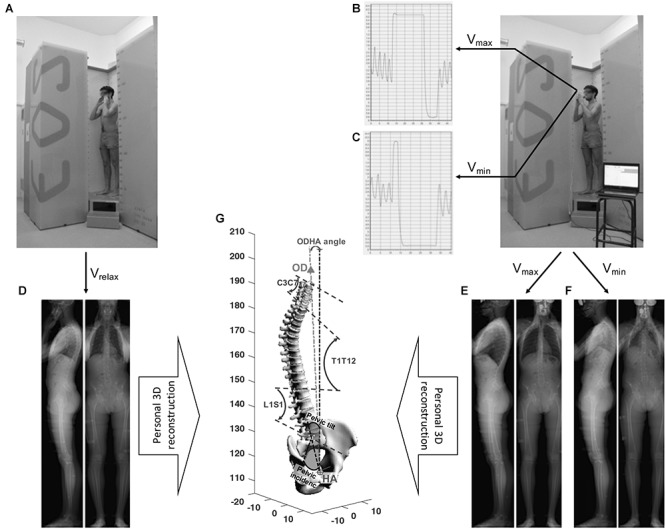
Experimental procedures. Low-dose, biplanar X-ray (BPXR) acquisitions at Vrelax in the standardized free-standing position **(A)** and using spirometry-controlled lung volume at Vmax **(B)** and Vmin **(C)**. Subject is fitted with a nose-clip and breaths through a mouthpiece connected to the spirometer. Spirometric curves are presented for Vmax **(B)** and Vmin **(C)**. BPXR acquisitions are done during breath holding. From frontal and sagittal radiological images at Vrelax **(D)**, Vmax **(E)**, and Vmin **(F)**, three 3D model specific are reconstructed (one model for each volume condition, but only one is represented in this figure) **(G)** including the odontoid process of C2 (OD), spine from C3 to S1 and pelvis. Then BPXR parameters are measured: C3–C7, T1–T12 and L1–S1 curvatures, pelvic variables, the angle between the vertical plane and the line through OD and the midpoint of the line connecting the center of the two femoral heads (HA) (ODHA).

Biplanar X-Rays (BPXR, see method next paragraph and Figure 1) were acquired during voluntary breath holding at relaxation volume (Vrelax, representing FRC), maximum inspiration (Vmax, intended as representative of TLC), maximum expiration (Vmin, intended as representative of RV). Changes in lung volumes between BPXR acquisitions were measured using a spirometer (low resistance pneumotachograph, M.E.C. PFT Systems Pocket-Spiro, Medical Electronics Construction, Brussels, Belgium), with the subjects wearing a nose-clip and breathing through a mouthpiece. The Vmax acquisition was performed after a maximal inspiration initiated from the end of a tidal expiration under steady-state conditions (stable tidal volume over several breathing cycles). During the acquisition (10 to 20 s depending on height), the subjects were verbally encouraged to maintain breath-hold while relaxing. After completion of the acquisition, the subject was asked to expire completely, then breathe quietly for several cycles, before disconnecting the spirometer. The Vmin acquisition was performed after a maximal expiration initiated from the end of a tidal expiration under steady-state conditions. The same procedure as for the Vmax acquisition was followed. IC and ERV were measured during the Vmax and Vmin acquisitions (IC*_bpxr_* and ERV*_bpxr_*, respectively) (see Figure 1).

### Biplanar Controlled Lung Volume Xrays of the Skeleton

The EOS^®^system (EOS^®^Imaging, France) is a low-dose biplanar x-ray system using sources placed at an angle of 90°, allowing simultaneous acquisition of frontal and sagittal radiological views of the whole skeleton (Dubousset et al., 2008). The first acquisition was performed at Vrelax, according to the procedure described by Amabile et al. (2016). Subjects were placed in the standardized free-standing position, with their hands placed on the cheek bone on each side of the face and breathed quietly during the acquisition. Then, BPXR acquisitions at Vmax and Vmin were then performed as described above. During these acquisitions, the position of the hands was slightly modified, as they were placed slightly more anteriorly in order to hold the spirometer. Of note, simulated acquisitions at Vmax and Vmin were performed before the actual acquisitions, to accustom the subject to the imaging procedure. The experimental plan is shown in Figure 1.

### BPXR Image Processing and Variables Recorded

A 3D model specific to each subject was constructed from frontal and sagittal radiological images. This model included the superior tip of the odontoid process of C2 (OD), the vertebrae from C3 to S1 and the pelvis. This model (see Figure 1) was produced by using validated reconstruction techniques (Mitton et al., 2006; Humbert et al., 2009). The following variables were calculated from this 3D reconstruction: (1) cervical (C3–C7), thoracic (T1–T12) (T4–T12) and lumbar (L1–S1) spinal curvatures, expressed in degrees; (2) pelvic variables (pelvic incidence, sacral slope and pelvic tilt); (3) the angle between the vertical plane and the line through OD and the midpoint of the line connecting the center of the two femoral heads (ODHA); (4) the anteroposterior distance between the vertical projection of C7 and the superolateral border of S1 (Sagittal Vertical Axis: SVA) (Amabile et al., 2016). OHDA characterizes the head-to-pelvis alignment (the smaller this value is, the closer the head-to-pelvis is to the vertical).

### Simulated Effects of Lung Volume Changes on Verticality in the Absence of Pelvic and Cervical Compensation

The following simulation was devised to illustrate what the impact of lung volume changes on posture would be in the absence of any pelvic and cervical spinal compensation: we reconstructed the whole pelvis-spine ensemble at Vmin and Vmax using the values of C3–C7 curvature and pelvic tilt by measured at Vrelax instead of their actual Vmin and Vmax values. This yielded “uncompensated values” of ODHA in the sagittal plane (ODHA_sagittalSimu_) and of SVA (SVA_simu_). We then confronted ODHA_sagittalSimu_ and SVA_simu_ at Vmin and Vmax to the range of values obtained for respectively, ODHA and SVA at Vmin and Vmax (considered as reference values in this group of subjects without any postural dysfunction), to estimate what would become of postural stability in the absence of cervical and pelvic compensation.

### Statistical Analysis

The distribution of most variables were non-normal, and all results are expressed as a median and interquartile range (Q1; Q3). To evaluate how Vmax and Vmin were representative of TLC and RV, respectively, IC_bpxr_ and ERV*_bpxr_* were compared to IC*_pft_* and ERV*_pft_* using Wilcoxon’s signed rank test for paired data. BPXR data obtained at Vmax and Vmin were separately compared to BPXR data at Vrelax by Wilcoxon’s signed rank test for paired data (no Vmin-Vmax comparisons). SVA was compared to SVA_simu_ and ODHA_sagittal_ was compared to ODHA_sagittSlsimu_ separately at Vmax and Vmin by Wilcoxon’s signed rank test for paired data. Associations between 1 age and BPXR parameters at the three volumes studied, ODHA_sagittalSimu_ and difference ODHA_sagittalSimu_-ODHA 2 dynamic lung volumes and T1–T12 curvature, were evaluated using Spearman’s correlation coefficient r_S_. All tests were two-tailed and *p*-values < 5% were considered statistically significant, except for the correlations with age which were adjusted with Benjamini–Hochberg’s correction for multiple testing with a target False Discovery Rate of 5%.

## Results

The complete protocol could be performed in the 50 subjects, but two subjects could not be analyzed due to BXPR acquisition issues. The following results therefore pertain to 48 subjects [22 women, age 34 (26; 48) years, Body Mass Index 23.6 (21.8; 25.9) kg/m^2^].

### BXPR Variables at Vrelax

The baseline characteristics of the subjects and all BPXR variables at Vrelax are presented in Table 1. Of note, at Vrelax, the median thoracic kyphosis T1–T12 was 52° (44; 59) and the median ODHA was 3° (2; 4). These values are within the range of previously published normal values (Amabile et al., 2016; Amabile et al., 2018).

**Table 1:** Baseline characteristics, pulmonary function tests and BPXR variables at Vrelax.

Baseline characteristics *N* = 48		
Gender M/F	26/22	
Age (years)	34 [26; 48]	
Height (m)	1.72 [1.65; 1.76]	
Weight (kg)	71 [62; 78]	
BMI (kg/m^2^)	23.6 [21.8; 25.9]	

**Pulmonary function tests reference values**		
	**L**	**% predicted**

VC_pft_	4.9 [4.0; 5.8]	113 [104; 126]
IC_pft_	3.3 [2.7; 4.0]	119 [106; 128]
ERV_pft_	1.6 [1.3; 1.9]	112 [99; 130]
FRC_pft_	3.3 [2.8; 3.7]	103 [96; 111]
RV_pft_	1.7 [1.3; 1.9]	87 [77; 102]
TLC_pft_	6.5 [5.6; 7.8]	106 [100; 117]

**BPXR parameters at Vrelax**		
Pelvic incidence (°)	51 [43; 60]	
Pelvic tilt (°)	12 [9; 16]	
Sacral slope (°)	-40 [-49; -34]	
ODHA_3D_ (°)	3 [2; 4]	
ODHA_sagittal_ (°)	-3 [-4; 1]	
ODHA_frontal_ (°)	0 [-1; 0]	
SVA (mm)	-7 [-23; 3]	
C3–C7 (°)	-5 [-10; 4]	
T1–T12 (°)	52 [44; 59]	
T4–T12 (°)	40 [32; 45]	
L1–S1 (°)	-58 [-69; -50]	


*BMI, body mass index; VC, vital capacity, IC, inspiratory capacity; ERV, expiratory reserve volume; FRC, functional residual capacity; RV, residual volume; TLC, total lung capacity; ODHA, odontoid-hip axis angle; SVA, sagittal vertical axis; C3–C7, cervical curvature between the third and seventh cervical vertebrae; T1–T12 and T4–T12, thoracic curvature between the first and twelfth and fourth and twelfth thoracic vertebrae, respectively; L1–S1, Lumbar curvature between the first lumbar vertebra and the sacrum*.

### Lung Volumes

Lung volumes from reference pulmonary function tests were available in the 48 subjects constituting the analysis population (Table 1). Spirometric data could only be obtained in 44 of these subjects during BPXR acquisitions (technical issues in 4 subjects). The difference between IC_pft_ and IC_bpxr_ was 0.40 (0.28; 0.74) L (*p* < 0.01). The difference between ERV_pft_ and ERV_bxpr_ was 0.24 (0.05; 0.45) L (*p* < 0.01). As a result, Vmax [6.1 (5.3; 7.1) L] represented 94% (89; 96) of TLC and Vmin [1.8 (1.4; 2.2) L] represented 113% (103; 127) of RV.

### Effects of Vital Capacity on Postural Alignment

**FIGURE 2:**
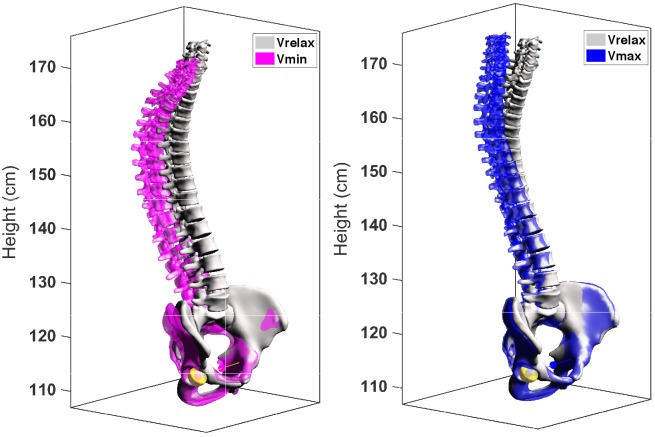
Specific 3D models of one subject at Vrelax Vmax and Vmin. Left: Vrelax (gray) and Vmin (purple) are superimposed. Right: Vrelax (gray) and Vmax (blue) are superimposed.

**FIGURE 3:**
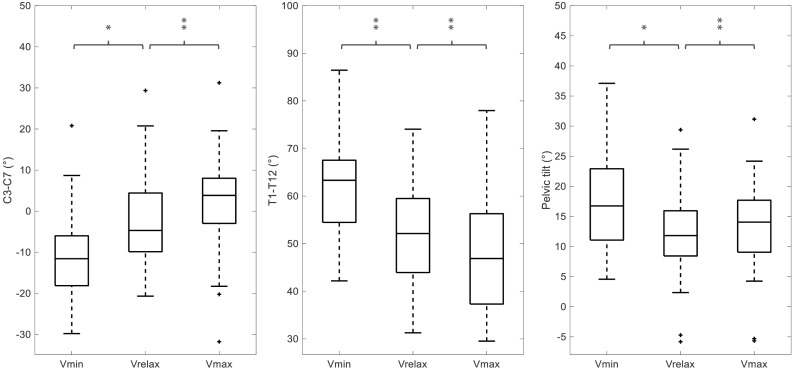
Influence of lung volume on BPXR parameters. C3–C7, T1–T12 and pelvic tilt values at Vrelax, Vmax, and Vmin in the 48 subjects. ^∗^*p* < 0.01 and ^∗∗^*p* < 0.001.

The detailed BPXR results are provided in Figure 2 and Table 2 (examples of 3D reconstructions in one subject), Figure 3, 4. Going from Vrelax to Vmax reduced thoracic kyphosis (median T1–T12 from 52° to 47°; *p* = 0.0007), reduced cervical lordosis (median C3–C7 from -5° to 4°; *p* = 0.006) and induced pelvic retroversion (median pelvic tilt from 12° to 14°; *p* = 0.002). Conversely, going from Vrelax to Vmin accentuated thoracic kyphosis (median T1–T12 from 52° to 63°; *p* = 8.96 × 10^-12^) and accentuated cervical lordosis (median C3–C7 from -5° to -12°; *p* = 6.70 × 10^-7^). It also induced pelvic retroversion (median pelvic tilt from 12° to 17°; *p* = 2.12 × 10^-9^). In both cases (Vrelax to Vmax and Vrelax to Vmin) the ODHA_3D_ angle was almost invariant (median variation of 1°).

**Table 2:** BPXR variables at Vmax and Vmin and their variations from Vrelax.

	Vmax	Difference	*p*	Vmin	Difference	*p*
Pelvic tilt (°)	14 [9; 18]	1 [0; 3]	0.002	17 [11; 23]	5 [1; 8]	2 × 10^-9^
Sacral slope (°)	-40 [-46; -34]	1 [-2; 4]	0.166	-36 [-43; -28]	6 [0; 9]	6 × 10^-7^
ODHA_3D_ (°)	4 [3; 6]	1 [0; 2]	1.18 × 10^-5^	4 [2; 6]	1 [0; 2]	0.014
ODHA_sagittal_ (°)	-4 [-5; -2]	-1 [-2; 0]	4.26 × 10^-6^	-3 [-5; -1]	0 [-1; 1]	0.418
ODHA_frontal_ (°)	0 [-1; 1]	0 [0; 1]	0.002	0 [-1; 1]	0 [0; 1]	0.006
SVA (mm)	-22 [-34; -10]	-11 [-23; -5]	9.49 × 10^-7^	-6 [-23; 9]	3 [-12; 21]	0.239
C3-C7 (°)	4 [-3; 8]	4 [-2; 11]	0.006	-12 [-17; -6]	-7 [-18; -1]	6 × 10^-7^
T1–T12 (°)	47 [37; 56]	-4 [-9; 1]	0.0007	63 [55; 68]	10 [5; 12]	8 × 10^-12^
T4–T12 (°)	33 [25; 40]	-5 [-9; 0]	1.18 × 10^-5^	47 [40; 53]	6 [2; 12]	1 × 10^-8^
L1–S1 (°)	-56 [-68; -51]	0 [-4; 5]	0.512	-57 [-66; -45]	3 [-1; 8]	0.012


*ODHA, odontoid-hip axis angle (3D: three-dimensional, sagittal: in the sagittal plane, frontal: in the frontal plane); SVA, sagittal vertical axis; C3–C7, cervical curvature between the third and seventh cervical vertebrae; T1–T12 and T4–T12: thoracic curvature between the first and twelfth and fourth and twelfth thoracic vertebrae, respectively; L1–S1, lumbar curvature between the first lumbar vertebra and the sacrum*.

**FIGURE 4:**
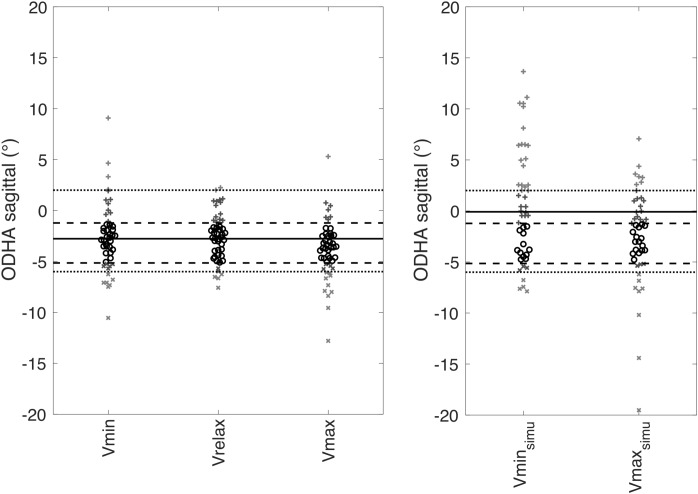
ODHA values at the three lung volumes and ODHA simulated values in the absence of pelvic and cervical compensation at Vmax and Vmin. On the left, representation of individual values of ODHA_sagittal_ at Vrelax (reference values), Vmax and Vmin. On the right representation of individual values of ODHA_sagittalSimu_ in the absence of pelvic and cervical compensation at Vmax (Vmax_simu_) and Vmin (Vmin_simu_). For both graphs, median of the reference values at Vrelax (in black solid line), 25–75th percentiles interval (in black dashed line) and normal values (Amabile et al., 2018) (in gray) are represented.

### Simulated Absence of Pelvic and Cervical Compensation (Figure 4, 5)

**FIGURE 5:**
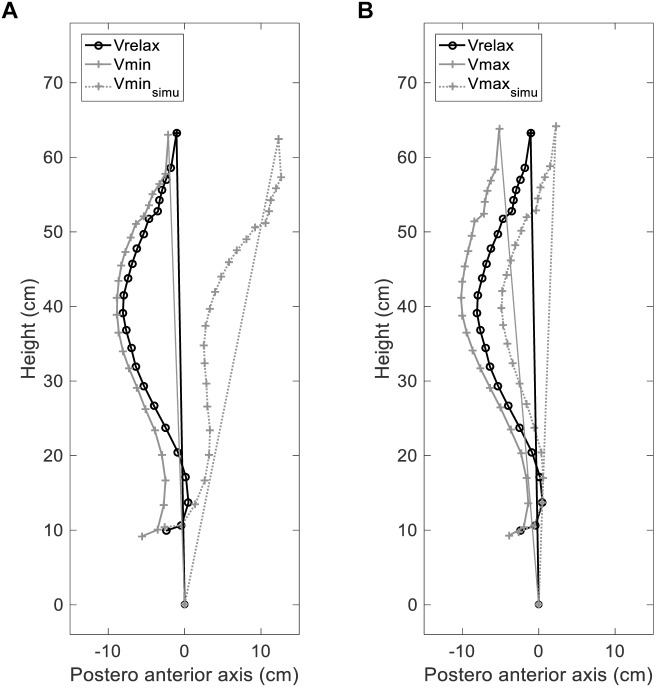
Simulated effects of lung volume changes on verticality. ODHA: angle between the vertical plane and the line through odontoid (OD) and the midpoint of the line connecting the center of the two femoral heads (HA); Vrelax: relaxation lung volume; Vmin: minimal lung volume; Vmax: maximal lung volume. **(A)** ODHA_sagittal_ angles in subject 10, measured at Vrelax (black solid line) (–1°) and Vmin (gray solid line) (–5°), and uncompensated (in the absence of pelvic and cervical compensation) simulated ODHA at Vmin (Vmin_simu_ gray dashed line) (+11°). **(B)** ODHA angles in the same subject, measured at Vrelax (black solid line) (–1°) and Vmax (gray solid line) (–5°), and uncompensated (in the absence of pelvic and cervical compensation) simulated ODHA at Vmax (Vmax_simu_ gray dashed line) (+2°).

Forty two subjects had ODHA_sagittal_ values in the normal range defined, previously in healthy subjects (Amabile et al., 2016, 2018) at Vrelax, 37 subjects at Vmin and 37 subjects at Vmax. In contrast, 31 subjects (65%) had ODHA_sagittalSimu_ values at Vmax and/or Vmin outside of the normal range. The differences between ODHA_sagittalSimu_ and ODHA_sagittal_ were 2 (0; 4) at Vmax (*p* = 0.001) and 4 (0; 7) at Vmin (*p* < 0.00001). Regarding SVA, the differences between SVA_simu_ and SVA were 7 (-6; 21) at Vmax (*p* < 0.0001) and 37 (7; 60) at Vmin (*p* < 0.0001).

### Correlations Between Lung Volumes, BPXR Values and Age

ERV*_bpxr_* correlated with the T1–T12 angle at Vmin (r_S_ = 0.3329; *p* = 0.027). Similarly, IC*b_pxr_* was correlated with the T1–T12 angle at Vmax (r_S_ = 0.3204; *p* = 0.034). In contrast, no correlation was observed between Vrelax and the T1–T12 angle at Vrelax (*p* = 0.154).

**FIGURE 6:**
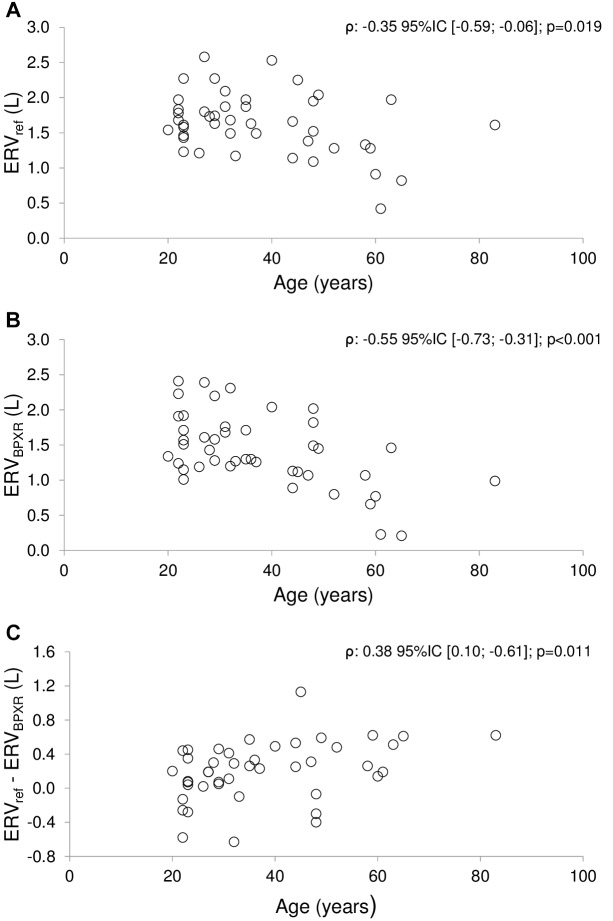
Correlations between age and expiratory reserve volume. ERV, Expiratory Reserve Volume; BPXR, Biplanar Xrays. **(A)** Correlation between age and expiratory reserve volume measured prior to the BPXR acquisition (spirometric reference values ERV_pft_). **(B)** Correlation between age and ERV measured during BPXR acquisition (ERV_bpxr_). **(C)** Correlation between age and the difference ERV_pft_ – ERV_bpxr_. rs: Spearman’s correlation coefficient; p: associated *p*-value; q: *p*-value adjusted with Benjamini–Hochberg’s correction for multiple testing.

Age was correlated with more marked pelvic tilt at Vrelax, Vmax and Vmin, a more marked C3–C7 lordosis at Vrelax and Vmax (see Table 3). In contrast, no correlation was observed between age and T1–T12, L1–S1 and ODHA at any of the three lung volumes studied and between age and ODHA_sagittalsimu_ at Vmax and Vmin. Age was correlated with lower ERV*_bpxr_* and a greater ERV*_pft_* – ERV*_bpxr_* difference (Figure 6). In contrast there was no correlation between age and neither ERV_pft_ nor IC (be it IC*_pft_*, IC*_bpxr_* or the difference between these values).

**Table 3:** Correlations between age and BPXR parameters.

	r_S_	*p*	*q*
			
Pelvic tilt at Vrelax	0.3748	0.00867366	0.03324904
Pelvic tilt atVmax	0.4608	0.00098087	0.01128
Pelvic tilt at Vmin	0.3949	0.00547981	0.03055895
C3–C7 at Vrelax	-0.3578	0.01252155	0.04114223
C3–C7 at Vmax	-0.3866	0.00664325	0.03055895


BPXR, biplanar X-ray; C3–C7, cervical curvature between the third and seventh cervical vertebrae; Vrelax, lung volume which corresponds to the functional residual capacity during BPXR acquisition; Vmax, lung volume after maximal inspiration during BPXR acquisition; Vmin, lung volume after maximal expiration during BPXR acquisition; r_*s*_, Spearman’s correlation coefficient; p: associated *p*-value; q: *p*-value adjusted with Benjamini–Hochberg’s correction for multiple testing.

## Discussion

This study shows that the changes in thoracic spinal curvature induced by maximal inspiration and maximal expiration in healthy humans, are fully compensated in terms of the head-to-pelvis alignment. This compensation is achieved through changes in the cervical spinal curvature and changes in the pelvic tilt. Simulations strongly suggest that in the absence of these cervical and pelvic compensations verticality could be compromised to the point of being responsible for falls.

### Methodological Strengths and Limitations

Respiratory-related changes in spinal curvature have been previously described (Dally, 1908), but this study is the first to describe postural adaptations over the range of vital capacity. It is also the first study to take advantage of the BPXR technology to address this issue. We obtained individual 3D skeleton models (Mitton et al., 2006; Dubousset et al., 2008; Humbert et al., 2009) at different lung volumes: this approach is known to allow a precise evaluation of postural alignment in weight bearing condition (Vialle et al., 2005; Amabile et al., 2016; Hasegawa et al., 2017). It has been validated in healthy subjects (Vialle et al., 2005; Amabile et al., 2016; Hasegawa et al., 2017) for the study of compensatory mechanisms during aging (Amabile et al., 2018), and in scoliotic patients (Ilharreborde et al., 2013).

We paid particular attention to perform BPXR acquisitions at reproducible lung volumes, hence the need to perform spirometric measurements during the procedure. The subjects therefore had to breathe into a spirometer and hold it themselves. Breathing through a spirometer has been shown to induce a postural constraint (Clavel et al., 2017). To limit the impact of this bias on our observations, we carefully instructed the subjects to hold the spirometer between the palms of their hands with their shoulders relaxed, in a position that was very close to the reference position (namely at relaxed end-expiratory lung volume, without the spirometer). We can however not rule out that the spirometric measurements interfered with our subjects’ standing posture. The amplitude of the corresponding changes, if any, was probably small respective to the postural modifications observed in response to the very large volume variations that we studied. Of note, Vmax and Vmin as measured during the BPXR acquisitions, significantly differed from TLC and RV. Nevertheless, the differences were small, and the dispersion of the values was limited.

As a limitation of the study, we acknowledge that our results pertain to postural adaptations to maximal lung volumes described under static rather than dynamic conditions. This limits their transposition to the study of respiratory-related postural perturbations and adaptations in real-life, particularly for hyperventilation, which is known to increase breathing-related postural perturbation (Hodges et al., 2002; Hamaoui et al., 2010; David et al., 2012) while holding the breath (as it was required in our study) is known to reduce or cancel it (Caron et al., 2004). In addition, nasal breathing maneuvers were not tested in these experiments for physiological comparison, as subjects were instructed to breathe through a mouthpiece. However, evaluations were done in a static condition, and consequently the spinal alignment we observed at extreme lung volumes was related to lung volume variations and was not influenced by the route of breathing before holding the breath.

### Postural Alignment and Vital Capacity

At extreme lung volumes, we observed variations of thoracic curvature resembling those long described during breathing (Dally, 1908) but of an expectedly greater amplitude. Indeed, inspiring to TLC and expiring to RV brought the T1–T12 angle outside its normal range (Vialle et al., 2005; Amabile et al., 2016) and resulted in highly variable SVA values. In the absence of compensation, this would compromise upright static balance (Roussouly et al., 2005; Vialle et al., 2005; Roussouly and Pinheiro-Franco, 2011b). Yet, in our subjects, verticality was preserved as ODHA values remained within the normal range (Amabile et al., 2016). We did observe compensations at the pelvic and lumbar level, as it is the case during resting breathing (Kantor et al., 2001; Caron et al., 2004; Hodges et al., 2007; Hamaoui et al., 2010; Talasz et al., 2011). We also observed the activation of a cervical compensatory mechanism (Figure 3 and Table 2). Combined pelvic and cervical compensations have been described during aging (Diebo et al., 2015; Amabile et al., 2018) and in degenerative diseases altering spinal posture (Roussouly and Pinheiro-Franco, 2011b; Diebo et al., 2015; Paternostre et al., 2017). They restore an adequate alignment of the head and pelvis to maintain horizontal gaze (Hasegawa et al., 2017) while achieving an energetically economical standing position (Duval-Beaupere et al., 1992). To our knowledge, cervical compensation has not been described before in the context of respiratory-related postural perturbations. This is possibly because the respiratory-induced changes in thoracic curvature during tidal breathing are not sufficient to trigger cervical adaptations, but become so when lung volume changes are very important as during our experiment (Scheer et al., 2013). In our subjects, changes in cervical spinal curvature appeared as a predominant postural compensatory mechanism, while the lumbar compensation was limited. The lumbar curvature is mostly determined by pelvic incidence, defined as the angle perpendicular to the sacral plate at its midpoint, and a line connecting the same point to the center of the bicoxofemoral axis. This angle is constant whatever the position; its values for one subject determine the global spinal alignment and particularly the magnitude of the lumbar lordosis, which partly explains the relatively low mobility of this spine segment (Roussouly et al., 2005). Moreover, inspiratory and expiratory efforts over the full range of vital capacity both induce increase of lumbar spinal stiffness, which in addition may limit lumbar mobility at extreme lung volumes (Shirley et al., 2003). This improvement in lumbar stiffness is due to an increase of trunk muscle activity and intra-abdominal pressure, and by the direct action of the diaphragm on the lumbar spine via its insertions (Shirley et al., 2003). Consequently, dramatic changes of thoracic curvature when maximally mobilizing lung volume, predominantly trigger cervical and pelvic segments, which are freer of motion (Roussouly and Pinheiro-Franco, 2011a).

In line with this, changes in lumbar curvature were small in our subjects both at Vmax (where they did not reach statistical significance) and at Vmin.

Moreover, the cervical lordosis is highly correlated to thoracic hyphosis when spine alignment varies (Shirley et al., 2003). In our subjects, it was adjusted to maintain the horizontal gaze when mobilizing vital capacity.

Simulations showed that in the absence of compensation, 65% of our subjects would have had ODHA values outside the normal range at either of the lung volumes studied, and therefore a compromised balance. Figure 5 clearly illustrates this phenomenon in one subject. Of note, maximum expiration induced more marked changes in thoracic spinal curvature (hyperkyphosis) than maximum inspiration. It also induced more intense pelvic postural compensation (retroversion) than maximum inspiration, suggesting that cervical compensations was less efficient during expiration than inspiration (Scheer et al., 2013). As a result, maximal expiration appears theoretically more threatening to postural stability than maximal inspiration.

This study fuels the notion that alterations in postural compensatory mechanisms involved by respiratory-related postural perturbations could constitute one of the determinants of the postural dysfunction observed during chronic respiratory diseases known to induce changes in lung volume or chest geometry, such as chronic obstructive pulmonary disease (COPD) (Janssens et al., 2014; Lahousse et al., 2015).

### Effects of Age

In our study population at Vrelax, age was significantly associated with more marked pelvic retroversion and more marked cervical lordosis. This is consistent with age-related postural adjustments previously reported (Diebo et al., 2015; Amabile et al., 2018). Of note, the postural adjustments that we observed during maximum expiration consist in an exaggeration of this pattern. Compensatory postural mechanisms during expiration could therefore be less effective in older people.

Normal aging is accompanied by decreased vital capacity with a decreased expiratory reserve volume -observed in our study population- and a decreased inspiratory capacity -not observed in our study population- (Turner et al., 1968). This is generally attributed to changes in thoracopulmonary mechanical properties (Verbeken et al., 1992; Galetke et al., 2007). The present data raise the hypothesis that the age-related reduction in vital capacity could partly proceed from deteriorated compensation of the respiratory-related postural perturbation. This is supported by the correlations observed in our subjects between lung volumes and T1–T12 curvature (increased kyphosis during expiration; decreased kyphosis during inspiration): greater lung volumes induce greater perturbations of postural alignment hence a greater need of postural compensation to maintain balance. This is also supported by the smaller IC and ERV values during BPXR acquisitions compared to reference values: although technical issues are possible, the subjects could have “censored” their respiratory efforts during BPXR acquisitions to preserve their balance. Of note, the differences between BPXR lung volumes and reference lung volumes were more marked with age (namely in subjects with lower postural compensation capacity) and particularly so for maximal expiration (that threatens balance more than maximal inspiration, see above). This postulated mechanism (impact of limited postural compensation capabilities on vital capacity) could, beyond aging, be called on to explain part of the impact of thoracic deformities on lung volumes [e.g., scoliosis (Szopa and Domagalska-Szopa, 2017) or secondary spinal lesions (Kenis-Coskun et al., 2017)].

## Conclusion

Extreme lung volume variations over vital capacity is associated with changes of thoracic curvature bringing it outside the normal range, which would theoretically compromise verticality. This is however fully compensated by adaptations of the cervical curvature and pelvic tilt to preserve adequate head-to-pelvis verticality and horizontal gaze alignment. Lung volume related postural perturbations increase with age, but age did not affect head-to-pelvis alignment. Future studies are needed to investigate potential postural dysfunction in chronic respiratory diseases that induce changes of lung volume or chest geometry, such as COPD.

## Ethics Statement

All subjects gave written informed consent in accordance with the Declaration of Helsinki. The protocol was approved by the Comité de Protection des Personnes Ile-de-France VI Paris (Ethics Committee).

## Author Contributions

VA, LC, BS, and TS contributed substantially to the study design, data analysis and interpretation, and the writing of the manuscript. WS, PR, IR, and SR-N contributed substantially to the data analysis and interpretation and to the writing of the manuscript.

## Conflict of Interest Statement

The authors declare that the research was conducted in the absence of any commercial or financial relationships that could be construed as a potential conflict of interest.
